# The impact of long-term school-based physical activity interventions on body mass index of primary school children – a meta-analysis of randomized controlled trials

**DOI:** 10.1186/s12889-016-2829-z

**Published:** 2016-03-01

**Authors:** Hong Mei, Yuelin Xiong, Shuixian Xie, Siyu Guo, Yukun Li, Bingbing Guo, Jianduan Zhang

**Affiliations:** Department Woman and Child’s Care and Adolescent Health, School of Public Health, Tongji Medical College, Huazhong University of Science and Technology, 13 Hangkong Rd., Wuhan, 430030 Hubei P.R. China

**Keywords:** Children, Obesity, Physical activity, School-based intervention, Effectiveness, Randomized controlled trail

## Abstract

**Background:**

Physical activity (PA) intervention is a commonly recommended strategy to combat childhood obesity. However, its effectiveness has long been controversial. This paper aims to examine the effectiveness of long-term (≥12 months) school-based PA interventions on body mass index (BMI) in primary school children, who are gaining BMI.

**Methods:**

Original papers were retrieved from PubMed, Google Scholar, the Cochrane Library and Web of Science, published between 1990 and 2015. The inclusion criteria were those research studies that were: randomized controlled trials (RCTs), conducted in primary school settings, had valid data on BMI at baseline and at the final follow up (or on BMI changes), and involved PA intervention that lasted for at least 12 months.

**Results:**

Out of 11,158 potentially eligible articles, 18 papers were included in the analysis, involving 22,381 primary school children with intervention durations ranging from 12 to 72 months. Compared to the control groups, the BMI increment was 2.23 kg/m^2^ less in the intervention groups (p < 0.05). The heterogeneity was high across the studies (99.8 %), but declined after sub-group analyses. The intervention type, intervention duration, and weekly PA intervention time were among the factors leading to the heterogeneity.

**Conclusion:**

Long-term school-based interventions containing PA as a core component appear to be effective in achieving healthier BMI. However, the results should be interpreted with caution due to the high heterogeneity among the studies. More high quality school-based RCTs among diverse populations are needed to improve the homogeneity and to yield a more robust conclusion.

## Background

The epidemic of childhood obesity has become a serious public health concern [1] due to its short and long-term physical and psychological consequences [2–4] and related economic burdens [5]. Results from the Global Burden of Disease Study 2013 indicated that in developed countries, 23.8 % of boys and 22.6 % of girls were overweight/obese; and in developing countries, the corresponding rates were 12.9 % and 13.4 % respectively [6]. Given the difficulties involved in weight loss [7], and the costly treatment of obesity [8], initiating obesity prevention at an early age has reached global consensus.

Regular physical activity (PA) established during childhood may lay the foundation for lifelong fitness [9]. Children with insufficient PA are at a higher risk to be overweight/obese and this risk increases with age [10]. Therefore, PA is considered as one of the most important obesity intervention strategies [11, 12]. To promote PA of children through the school setting has its merits compared to the household and the community environment [13–15], because children are better organized and can be collectively reached at school [16, 17]. As all individuals are at risk of gaining extra weight [18], school-based PA intervention should target all students regardless of their weight status. This also avoids the possible stigma caused by only putting the overweight/obese students into the spotlight [19].

Several studies have reported the impact of school-based PA interventions on students’ obesity problems, yet failed to yield consistent conclusions [20–24]. Recently research revealed that school-based PA interventions were ineffective on reducing the BMI increment in primary school students [25], while some others held the opposite conclusion [26, 27]. The inconsistency of the conclusions may result from the variation in study characteristics (study population, age, gender, and sample size), study design, intervention duration, strategies, etc. For instance, the TrimTots programme, involving randomized controlled trials (RCTs) of PA interventions in preschool and primary school age children, indicated a significant reduction in obesity risk after a long term follow-up [28]; while the results from the ECLS-K study, an earlier childhood longitudinal study in primary school, demonstrated that physical education has no significant impact on BMI if the students had a normal weight/overweight status [29]. A recently published systematic review by the Cochrane Collaboration demonstrated the positive impact of school-based PA interventions on students’ behavior and on physical measurements [30]. However, since the studies used in the review possessed wide spans in the participants’ age (from 6 to 18 years) and in the intervention duration (from 12 weeks to 6 years), the authors suggested the results should be interpreted with caution. In addition, Paulo et al also suggested that the impact of PA programs in promoting fitness of children should not be generalized because of the variation in duration of the PA programs and intensity and type of PA among studies [31].

Furthermore, in consideration of the out-of-school impact, e.g., holiday recess might compromise the effects from school days [32], and because fostering healthy lifestyle habits is a long-term process [33, 34], we suspect that long-term PA interventions directed at young students may yield more robust and convincing results. In the present meta-analysis, with stringent inclusion criteria, we aimed to more precisely and holistically understand whether long-term school-based RCT PA interventions could benefit primary school children’s growth; and a further aim was to investigate if the study area, design and quality, intervention duration, weekly PA intervention time, PA intervention type, and measurement could lead to the significant benefits.

## Methods

### Literature search

Keywords in English, including physical activity, physical education, exercise or active break, body mass index or BMI or obesity, and school children were used individually or in combination to retrieve related articles published between January 1990 and March 2015 from major databases (PubMed, Web of Science, the Cochrane Library and Google Scholar). The retrieve protocol was like ((((physical activity [MeSH Major Topic]) OR physical education [MeSH Major Topic]) OR exercise [MeSH Major Topic]) AND body mass index [MeSH Major Topic]). The bibliographies of relevant meta-analysis and systematic reviews were also manually investigated to retrieve additional relevant original articles that met the inclusion criteria indicated as follow. The preliminary retrieved references were carefully examined to avoid duplications and omissions.

### Inclusion criteria

The analysis was limited to the studies published in English and with human subjects. The following criteria were used for paper selection: 1) primary school-aged children (6 to 12 year-old) regardless of their weight status, 2) RCT designed, 3) intervention conducted in the school setting, 4) PA intervention duration ≥12 months, 5) available data on the mean and standard deviation (SD) of BMI or the BMI changes from baseline to the final follow-up in both intervention and control groups.

### Data extraction and validity assessment

The initial screening of titles and abstracts was performed by one investigator with a randomly selected 10 % of the sample checked by a second investigator. Data were independently extracted by one investigator using a self-made data-collection form developed based on the inclusion criteria and was independently checked by a second investigator. The following information of studies including basic information (authors, publishing year, and study area), study design and sample size, grade and gender of students, intervention type and duration, and PA measurement were extracted from the eligible studies. Another two investigators conducted internal and overall validity assessments independently using the Jadad Scale scoring algorithm [35]. The sum of the scores of all five items forms the final Jadad Scale score (JSS) for each study, ranged from 0 to 5. Studies with JSS equal to or greater than 3 were considered as high quality studies. If the score for the same item differed between the two investigators, a third investigator had to reassess the study and at last come to an agreement on all the items of the included studies. The JSS has been used in previous RCT studies [36].

### Data synthesis and analysis

The eligible studies were originated in European, Asian, and African countries and in the United States. The study’s design was RCT or cluster RCT with randomization at the school level. The intervention type was stratified as physical activity only (PA) and physical activity plus nutrition (PA&N). Intervention duration was presented in months as a dichotomous variable (12 ~ 24 months and >24 months). PA level was electronically monitored and/or collected by using questionnaires, therefore the measurement was classified as electronic instrument only (I), questionnaire only (Q) or both (I & Q). PA contents detailed the frequency and duration of weekly PA at school. Weekly PA intervention time was calculated as the original physical education time plus the additional PA intervention time, presented as minutes per week (min/week) and was classified as ≤100 min/week and >100 min/week. The final BMI was measured immediately after the completion of the intervention. The Chi-square test was used to compare the difference of JSS between RCT studies and cluster RCT studies.

The primary analysis was focused on the long-term effect of PA interventions on the BMI of primary school children. The mean change in BMI (^△^BMI) for both intervention groups (IG) and control groups (CG) was calculated as BMI at final follow up minus BMI at baseline. For each study, the effect size was the difference in ^△^BMI between control and intervention groups (^△^BMI_IG_-^△^BMI_CG_) and then was expressed as the standardized mean difference (SMD) with a 90 % confidence interval (CI). A crude forest plot was conducted to graph the effect size of studies on **△**BMI using random effect model. In the forest plot, the SMD of each study was displayed, along with a combined estimated variance of the overall effect. *I*^*2*^ was used to assess the heterogeneity of SMD across the included studies, while Begg’s funnel plotting was adopted to visualize the publication bias. Thereafter, one-way sensitivity analysis was performed to assess the robustness of the results by removing each study individually and assessing the *I*^*2*^ impact on the summary estimate. After removing the studies that highly impact the summary estimate, stratified analyses were used to detect the contribution of study design, origination area, duration, intervention type, PA measurement method, and weekly PA intervention time on the origin of heterogeneity.

All statistical analyses were performed using STATA version 12.0.

## Results

### Literature eligibility

**Fig. 1:**
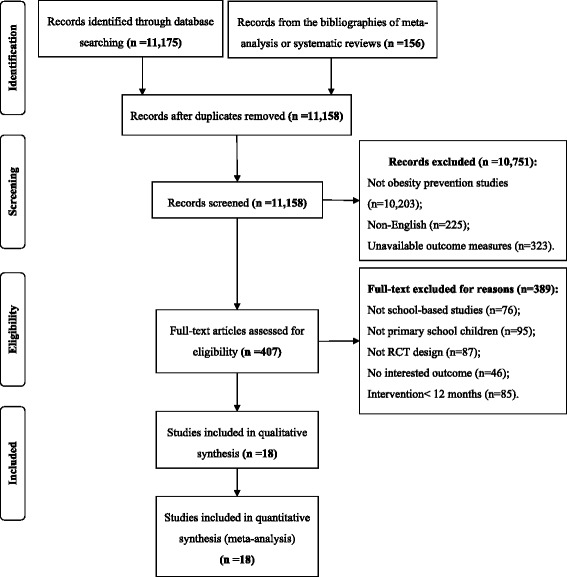
Flow Diagram of article research

There were 11,175 potentially relevant articles identified according to the search protocol, and another 156 articles were manually retrieved from the reference lists of relevant meta-analysis and systematic reviews. With duplicates of records searched in both search protocol and manually retrieved, there was 11,158 left. Of those, 10,751 studies were excluded for being non-obesity prevention studies (10,203 records), non-English (225 records), and without available outcome measures (323 records). An additional 389 studies were excluded due to the following reasons: non-school-based studies (76 records), not from primary school (95 records), non-RCT (87 records), no BMI outcome available (46 records), and intervention duration <12 months (85 records). As a result, 18 eligible studies were retained for the meta-analysis. The flow chart is shown in Fig. 1.

### Characteristics of included studies

The 18 studies were published from 1999 to 2014, with a total of 22,381 children included. Two of the included articles were from the same research project (one presented the medium term effect and one showed the final term effect) [37, 38]. Nine studies were conducted in Europe [33, 39–46], 5 studies were from the United States [34, 47–50], 4 studies were conducted in Africa [37, 38] and Asia [51, 52] (2 each). Among the 18 studies, 7 studies (38.9 %) included more than 1000 children; 3 (16.7 %) studies have only recruited roughly 100 children, the rest ranged from 346 to 646 children. Most of the included studies targeted both genders with a gender ratio (boy to girl) that ranged from 0.79 to 1.18; while only one targeted boys. Five interventions (27.8 %) lasted less than two years, and 8 of them (44.2 %) continued for at least three years. The majority of the studies (14, 77.8 %) were cluster RCT, and the reminding 4 (22.2 %) studies were RCTs. Five studies (27.8 %) only included PA intervention and the rest 13 (72.2 %) involved both PA and nutrition components. For the measurement method of PA, 11 studies (61.1 %) only used questionnaires, 5 (27.8 %) adopted electronic instruments, and 2 study (11.1 %) used both questionnaires and electronic instruments. There were 8 studies that indicated PA intensity indicators. Both Mackelvie et al. [39] and Ahamed et al. [43] used the scores from Physical Activity Questionnaires (PAQ-C) for Children to assess PA intensity, while only Ahamed et al. [43] stressed the PAQ-C scores for both baseline and terminal line. The other two studies from Manios et al. [33] and Angelopoulos et al. [41] used the same PA intervention and same questionnaire to assess the moderate-to-vigorous PA (MVPA). While the former one shown significant effect of MVPA intervention on reducing BMI increment, the latter one shown nonsignificant results. There were 4 studies that used electronic instruments to assess PA intensity. The instruments differed between these studies (Caballero et al. [47] used the Tritrac R3D, Hemokinetics, Iowa City; Donnelly et al. [48] used Actigraph, 7163, Pensacola, FL; Dzewaltowski et al. [50] used Actigrap GT1M accelerometers, Shalimar, FL; Kriemler et al. [40] used MTI/CSA 7164, Actigraph, Shalimar, FL). There were 12 studies conducted with the PA intervention time ≤100 min/week and 6 studies with PA intervention time ranged from 120–450 min/week. Details can be seen in Table 1.

**Table 1:** Characteristics of the 18 studies in the meta-analysis

Study	Country	Study design	Gender (M/F^*^)	Age (grade)	Baseline sample size (N; IG/CG^*^)	Final sample size (N;IG/CG)	Intervention type	Intervention duration(month)	Measurement method of PA level	PA contents	Weekly PA intervention time (minute)
Aguilar,2010 [44]	European	Cluster RCT	MF(0.94)	4 ~ 5	921;375/546	546;257/289	PA	12 ~ 24	I	Three 90-minute PA sessions/week, and three hours low-to-moderate intensity PA sessions/week	>100
Ahamed,2007 [43]	European	Cluster RCT	MF(0.99)	4 ~ 5	288;214/74	287;214/73	PA&N	12 ~ 24	Q	80 min/week physical education and 75 min/week PA in the classroom	>100
Angelopoulos,2009 [41]	European	Cluster RCT	MF(0.79)	1 ~ 6	646;321/325	646;321/325	PA&N	12 ~ 24	Q	Two 45-minute PA sessions/week.	≤100
Caballero,2003 [47]	The United States	RCT	MF(-)	3 ~ 5	1704; 879/825	1409; 727/682	PA&N	>24	I&Q	Three 30-minute PA sessions/week	≤100
Donnelly,2009 [48]	The United States	Cluster RCT	MF(0.94)	2	1527;814/713	1427;792/698	PA	>24	I	90 minutes moderate-to-vigorous intensity PA sessions/week	≤100
Dzewaltowski,2010 [50]	The United States	Cluster RCT	MF(0.98)	3 ~ 4	246;112/134	246;112/134	PA&N	>24	I	Five 30-minute PA sessions/week	>100
Graf,2008 [45]	European	Cluster RCT	MF(1.04)	1	580;410/170	580;410/170	PA&N	>24	Q	Extra 20–30-minute/week health education and 5-minute/day PA games	≤100
Jiang,2007 [52]	Asian	Cluster RCT	MF(1.06)	3 ~ 6	2452;1029/1396	2452;1029/1396	PA&N	>24	Q	Three 20-minutes/week running	≤100
Kriemler,2010 [40]	European	Cluster RCT	MF(0.95)	1, 5	502;297/205	502;297/205	PA	12 ~ 24	I	Two 45-minutes PA sessions/week	≤100
Li,2010 [51]	Asian	Cluster RCT	MF(1.04)	3 ~ 4	4120;2092/2028	4120;2092/2028	PA	12 ~ 24	I&Q	Ten 10-minutes PA sessions/week	≤100
Llargues,2011 [37]	African	Cluster RCT	MF(1.18)	1	509;272/237	508; 272/236	PA&N	12 ~ 24	Q	Three-hour activities related to PA/week	>100
Llargues,2012 [38]	African	Cluster RCT	MF(1.09)	1	426;225/201	426;225/201	PA&N	12 ~ 24	Q	Three-hour PA/week	>100
Lohman,2003 [49]	The United States	RCT	MF(1.08)	-	1367;704/663	1367;704/663	PA&N	>24	Q	Three 30-minute PA/week	≤100
MacKelvie,2004 [39]	European	RCT	M	4 ~ 6	64;31/33	64;31/33	PA	12 ~ 24	Q	Three time 12-minute PA/week	≤100
Magnusson,2012 [42]	European	Cluster RCT	MF(0.80)	2	166;90/76	166;90/76	PA&N	12 ~ 24	I	Three 40-minute PA sessions/week	>100
Manios,2002 [33]	European	RCT	MF(-)	1	1046;602/444	641;356/285	PA&N	>24	Q	Two 45-minute PA sessions/week	≤100
Nader,1999 [34]	The United States	Cluster RCT	MF(1.07)	3 ~ 5	5106;2989/2117	4544;2707/1837	PA&N	>24	Q	Two 45-minute PA sessions/week	≤100
Tarro,2014 [46]	European	Cluster RCT	MF(-)	2 ~ 3	2350;1550/800	1939;1222/717	PA&N	>24	Q	12 hour PA and nutrition class per academic school	≤100

*M* male, *F* female, *N* total number of simple size, *IG* intervention group, *CG* control group, *PA* physical activity intervention, *PA&N* physical activity and nutrition intervention; -: unclear

### Quality of the studies

Two investigators individually assessed the quality of 18 studies using the Jadad Scale and yielded almost identical JSSs, i.e., only 6 items were scoring differently out of 126 items. A third investigator reexamined the eligible studies on each item especially the 6 inconsistent items, and then the final JSS were given to each study. Five studies received scores of 3, 12 studies attained scores of 2, and the remaining one received 1 score. No significant difference in quality was found between RCTs and cluster RCTs by the Chi-square test. Results were presented in Table 2.

**Table 2:** Jadad score assessing the quality of the 18 studies

Study	Was the study described as randomized?	Was the method used to generate the sequence of randomization described and appropriate?	Deduct one point if the method used to generate the sequence of randomization was described and it was inappropriate.	Was the study described as double blind?	Was there a description of withdrawals and dropouts?	Was the method of double blinding described and appropriate?	Deduct one point if the study was described as double blind but the method of blinding was inappropriate.
Aguilar,2010 [44]	1	1	0	0	1	0	0
Ahamed,2007 [43]	1	0	0	0	1	0	0
Angelopoulos,2009 [41]	1	1	0	0	0	0	0
Caballer,2003 [47]	1	1	0	0	1	0	0
Donnelly,2009 [48]	1	0	0	0	1	0	0
Dzewaltowski,2010 [50]	1	1	0	0	1	0	0
Graf,2008 [45]	1	0	0	0	1	0	0
Jiang,2007 [52]	1	0	0	0	1	0	0
Kriemler,2010 [40]	1	1	0	0	1	0	0
Li,2010 [51]	1	0	0	0	1	0	0
Llargues,2011 [37]	1	0	0	0	1	0	0
Llargues,2012 [38]	1	0	0	0	1	0	0
Lohoman,2003 [49]	1	0	0	0	1	0	0
MacKelvie,2004 [39]	1	1	0	0	1	0	0
Magnusson,2012 [42]	1	0	0	0	0	0	0
Manios,2002 [33]	1	0	0	0	1	0	0
Nader,1999 [34]	1	0	0	0	1	0	0
Tarro,2014 [46]	1	0	0	0	1	0	0

### Primary outcome

**Fig. 2:**
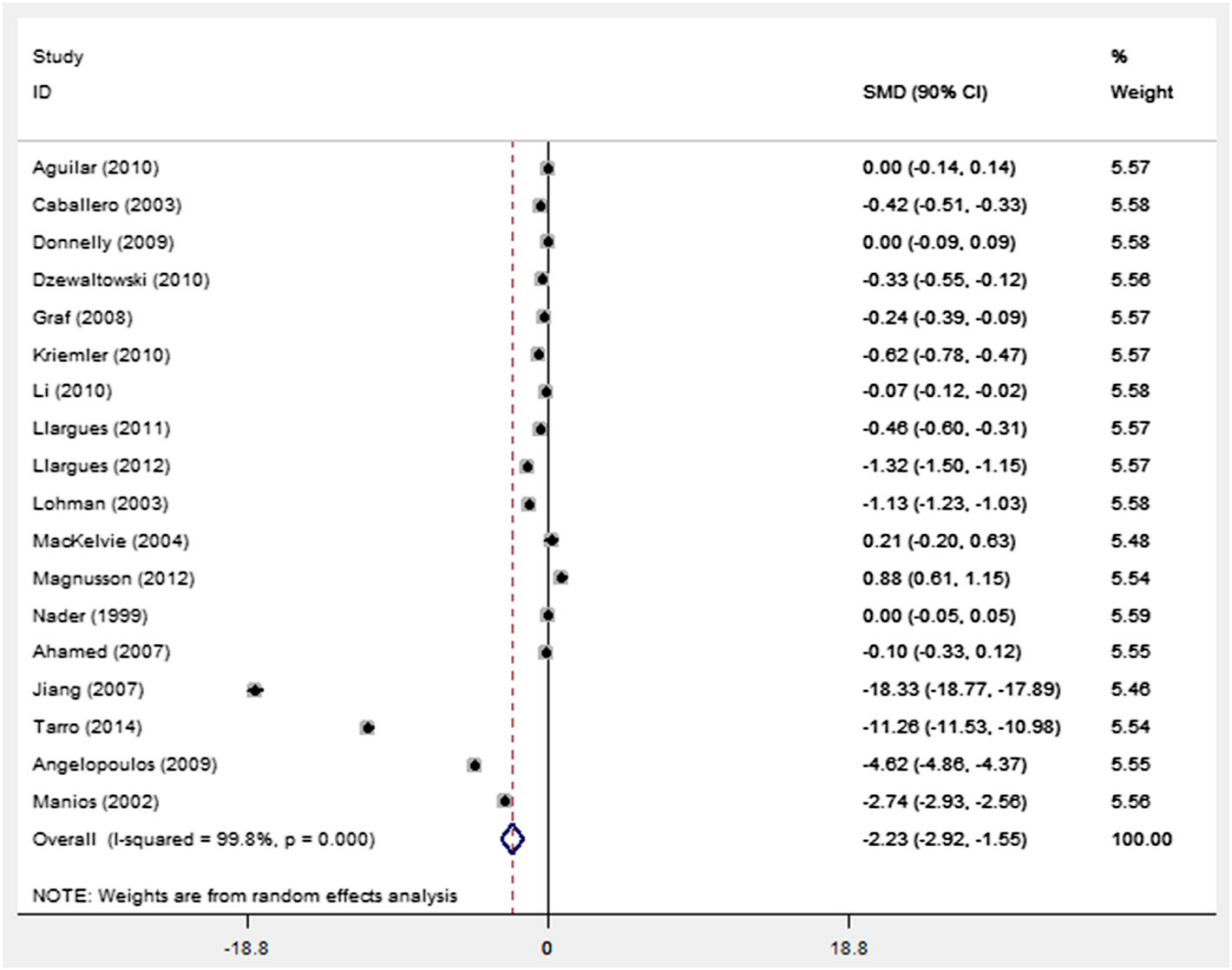
The forest plot for the 18 studies by publishing year. The filled triangles and diamonds represent the SMD and 90 % confidence interval for each study with a default weight percentage. The diamond with hollow refers to the overall SMD and 90 % CI, along with the vertical dashed line as centerline of the average SMD for the 18 studies. Random effect was used for the analysis

**Fig. 3:**
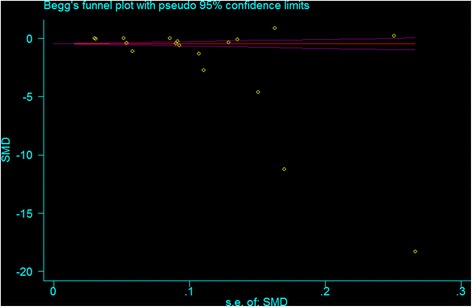
The funnel plot for the 18 studies. The horizontal axis is the coefficient of SMD for BMI change in intervention groups and control groups, and vertical axis (s.e.of: SMD) is the reciprocal of SMD for BMI change in intervention groups and control groups. Random effect was used for the analysis

Children’s ^△^BMI was significantly different (*p* < 0.05) between the PA intervention group and the control group (SMD: -2.23 kg/m^2,^ 90 % CI: -2.92, -1.56) (Fig. 2). High heterogeneity (*I*^*2*^ = 99.8 %) was identified cross the studies. The Begg’s Funnel plot was asymmetric with some outliers (*p* < 0.05) (Fig. 3).

### Sensitivity and stratified analysis

The meta-influence plot showed 7 studies (from Jiang et al. [52], Tarro et al. [46], Angelopoulos et al. [41], Manios et al. [33], Llargues et al. [38], Lohman et al. [49], and Magnusson et al. [42]) have significant impacts on the overall effect. When these studies were removed from the analysis, the difference of ^△^BMI between the intervention group and the control group increased from -2.23 kg/m^2^ to -2.00 kg/m^2^ (90 % CI: -0.30, -0.09). Simultaneously, the heterogeneity among the remaining studies was reduced (*I*^*2*^ = 90.5 %).

With the seven studies excluded, stratified analysis showed that intervention type (PA and PA&N) did affect the summary estimate of ^△^BMI. The difference of **△**BMI for PA group and control group was -0.13 (N = 5, 90 % CI: -0.29, 0.04; *I*^*2*^ = 89.8 %); while ^△^BMI between PA&N group and control group was -0.26 kg/m^2^ (N = 6, 90 % CI: -0.30, -0.09; *I*^*2*^ = 92.1 %). The SMD for studies with the weekly PA time ranged from 36–100 min was -0.22 (90 % CI: -0.38, -0.05, *I*^2^ = 94.0 %), and for weekly PA interventions >100 min, the SMD was -0.18 (90 % CI: -0.35, 0.00; *I*^2^ = 87.1 %). Overall intervention duration of studies also impacted the BMI change. The difference of ^△^BMI was significantly different between the interventions that lasted for 12–24 months groups and control groups (SMD: -0.20 kg/m^2^, 90 % CI: -0.39, -0.02); and the difference of ^△^BMI in intervention lasted for over two years and the control group was -0.19 kg/m^2^ (90 % CI: -0.35, -0.03).

Stratified analysis specific to the study area, design, and quality showed no significant difference in BMI changes between different groups. No group difference was found for PA measurement (*p* > 0.05).

## Discussion

The impact of school-based PA interventions on obesity is inconsistent across studies [20, 53–55]. In the present meta-analysis of 18 studies, we found an overall significant impact of long-term PA intervention on reducing BMI gain among primary school children (2.23 kg/m^2^ greater in the intervention group than in the control group). Therefore, long-term PA intervention should continue to be stressed as one of the core strategies in battling childhood obesity.

In our study, the PA&N intervention durations that lasted for at least one year had a positive impact on children’s ^△^BMI. Several reviews shared the same conclusion as our analysis [56–59]. Lavelle et al concluded in a recent review, when the intervention duration of the studies involved ranged from 1 to 72 months, that school-based PA interventions are effective in promoting healthier BMI among children under 18 years old [60]. A synthesis analysis of existing systematic reviews and meta-analysis from Khambalia et al also stated that PA intervention lasting for over one year, especially combined with diet intervention, significantly reduced body weight in children [59]. However, the similar trends were not always found in some other reviews [30, 61–64]. In a meta-analysis with a total of 18,141 children included and the interventions lasting for a minimum of six months, Harris et al. declaimed that school-based PA interventions do not improve body composition [64], although PA is one of the key components of a healthy lifestyle and contributes to many aspects of health [65]. Despite the positive effect of PA interventions on physical activity behaviors, i.e., increased MVPA and prolonged time students spent on PA, Lonsdale et al declared in his review paper that PA interventions had little effect on BMI [63].

In our results, weekly PA intervention for both ≤100 min and >100 min reduced children’s BMI increment significantly, while the reduction of BMI increment was higher in the group with intervention ≤100 min. A common sense would be an intervention introducing longer time PA should be more effective on reducing BMI increment. However this was not supported by our results. Our results, in addition to indicate the shorter PA time the better, tended to indicate that a well-designed intervention program should be implementable as well as scientifically sounds. An intervention simply using long PA time as the intervention strategy might result in a poor implementation, and then lead to poor outcome. To improve the interest and spirit of children’s attitude to get a higher intensity and longer duration of MVPA in primary school children, more appropriate and enjoyable PA interventions involving a variety of activities are needed [66, 67].

The large variation in characteristics of participants, such as a wide age span (6 to 18 years), large range of intervention duration (from 1 month to 6 years), and PA intervention intensity (from additional 4-min walking/running per day to 3-h MVPA) not only led to the inconsistent conclusions regarding the impact of PA intervention across the studies, but could also hinder the discovery of the real impact. For example, the impact of the long-term intervention on BMI, if any, might be comprised by those studies with short intervention duration, as the significant reduction of BMI is unlikely to happen in a short-term. This reinforces the importance of investigating the effect of long-term PA intervention on BMI among children with a narrower age span, i.e., elementary students, in order to yield a more promising conclusion.

Along with the strict inclusion criteria in our analysis, a high overall heterogeneity in the recruited studies was observed and is considered as one of the limitations of the study. Seven studies identified by sensitivity analysis have the greatest impact on the overall BMI change [33, 38, 41, 42, 46, 49, 52], with the BMI SMD as -2.23 kg/m^2^ (90 % CI: -2.92, -1.56) for the 18 studies and -2.0 kg/m^2^ (90 % CI from -0.30, -0.09) for the remaining 11 studies. Some characteristics of the seven studies, including study duration, intervention type, PA duration, age range, sample size, year conducted, and geographic area accounted for the high heterogeneity. For instance, Angeloupoulos et al had the shortest intervention duration (12 months), a relatively short PA duration (60 min/week) but the widest age range (from grade one to six) [41]; studies from Manios et al were conducted in early 90’s which is about one decade earlier than the rest of the studies [33], when the obesity problem had not yet become as serious as it is now [68]. The sample size of Magnusson et al. [42] is only 166 and Llargues et al. [38] is the only one that was conducted in an African country. These variations could also lead to the high heterogeneity. In the study of Lohman et al. [49], the details regarding the age range of included children and the measurement of PA level were not available, which may also contribute to the heterogeneity among studies. As a result, multiple factors, such as the age of participants, the type of intervention and the duration, sample size, socio-economic variables, etc., collectively contributed to the high heterogeneity. After the exclusion of the seven studies, the heterogeneity was reduced but still remained relatively high, which can also be found in some former synthesis of systematic reviews and meta-analysis [59]. Analysis from Shijun Li suggested that *I*^2^ was only suitable for testing heterogeneity amongst small sample size trials [69]. As the current meta-analysis involved more than 19,700 children, *I*^2^ might not be an appropriate variable to assess heterogeneity in this study.

Another limitation of the analysis was about PA intensity. It would be more straightforward if a recommendation regarding the best practice of PA intensity could be provided. Unfortunately, as the included studies used inconsistent measurements or definition of PA intensity (e.g., different questionnaires or different electronic equipment were used to collect the data about PA intensity), we were unable to specify the PA intensity in our analyses. This limitation also existed in previous studies, too [11]. Further work is needed to establish more comparable PA measurement standards that can be used in different studies.

The asymmetric funnel plot indicated that studies included in our analysis may have publication bias. This may be due to the publication preference that reports with positive or significant outcomes are more likely to be published or reported [70]. The low quality of included studies (low JSS) was also of concern in the current analysis.

Reduced level of PA and increased sedentary lifestyles have greatly contributed to the rapid increase of childhood obesity prevalence [71]. The competitive society and score-oriented education strategy and long school hours have further edged out the exercise time of school-age children [72]. For example, although the Chinese government has initiated a recommendation that students should achieve at least 60 min of after-class PA per day [73], only 14.5 % of the students reached this goal in 2011 [74]. In the United States and Canada, children and adolescents are also falling short of benchmarks for PA and fitness [75, 76]. Our results reinforce the importance of PA interventions in the school settings to battle the prevalence of childhood obesity. More venues for PA exercises and more feasible and attractive curricular and extracurricular physical exercises should be made available to ensure the quality and quantity of PA among school children. How to effectively implement the school PA, policy should be carefully determined.

## Conclusions

The meta-analysis tends to support the effectiveness of long-term PA intervention on promoting healthier BMI among children in primary school settings. The high heterogeneity of recruited studies suggests more high quality school-based RCTs among diverse populations are needed to yield a more robust conclusion.

## Abbreviations

**△**BMI: BMI change from the baseline to the final follow up
**△**BMI_CG_: BMI change in the control group
**△**BMI_IG_: BMI change in the intervention group
BMI: body mass index
CG: control group
CI: confidence interval
I: instrument only
I&Q: instrument and questionnaire
IG: intervention group
MVPA: moderate-to-vigorous physical activity
N: number of included studies
PA: physical activity
PA&N: physical activity and nutrition intervention
Q: questionnaire only
RCTs: randomized controlled trials
SD: standard deviation
SMD: standardized mean difference
